# One to Rule Them All: A Unique TAU Therapy for Neurodevelopmental Encephalopathies

**DOI:** 10.1177/15357597221126332

**Published:** 2022-09-22

**Authors:** Benito Maffei, Gabriele Lignani

**Affiliations:** 1 Department of Clinical and Experimental Epilepsy, Queen Square Institute of Neurology, University College London, United Kingdom

## Abstract

**TAU Ablation in Excitatory Neurons and Postnatal TAU Knockdown Reduce Epilepsy, SUDEP, and Autism Behaviors in a Dravet Syndrome Model**

Shao E, Chang C-W, Li Z, Yu X, Ho K, Zhang M, Wang X, Simms J, Lo I, Speckart J, Holtzman J, Yu G-Q, Roberson ED, Mucke L. *Sci Transl Med.* 2022;14(642):eabm5527. doi:10.1126/scitranslmed.abm5527

Intracellular accumulation of TAU aggregates is a hallmark of several neurodegenerative diseases. However, global genetic reduction of TAU is beneficial also in models of other brain disorders that lack such TAU pathology, suggesting a pathogenic role of nonaggregated TAU. Here, conditional ablation of TAU in excitatory, but not inhibitory, neurons reduced epilepsy, sudden unexpected death in epilepsy, overactivation of the phosphoinositide 3-kinase-AKT-mammalian target of rapamycin pathway, brain overgrowth (megalencephaly), and autism-like behaviors in a mouse model of Dravet syndrome, a severe epileptic encephalopathy of early childhood. Furthermore, treatment with a TAU-lowering antisense oligonucleotide, initiated on postnatal day 10, had similar therapeutic effects in this mouse model. Our findings suggest that excitatory neurons are the critical cell type in which TAU has to be reduced to counteract brain dysfunctions associated with Dravet syndrome and that overall cerebral TAU reduction could have similar benefits, even when initiated postnatally.

## Commentary

Dravet syndrome (DS) is a severe epileptic encephalopathy characterized by seizure activity and cognitive deficits early in childhood. With the major cause being heterozygous loss-of-function mutations of the *SCN1A* gene, coding for the voltage gated sodium channel Nav1.1, therapeutic strategies aimed at restoring gene function are usually sought.^1^ Despite TAU being best characterized as a microtubule-associated protein, it is well-known to have significant influence on intrinsic neuronal excitability, and therefore seizures, in both pathological and nonpathological forms. It is also well documented that reducing endogenous TAU decreases hyperexcitability induced by proconvulsant, limits seizure severity, and increases seizure latency in chemically induced acute seizures in healthy animals, and reduces seizure frequency in mouse models of genetic epilepsies.^2-4^

In prior reports, the authors have confirmed that TAU reduction prevents various aspects of DS^5^ in an animal model and that the combination of effects on excitatory and inhibitory neurons in vitro counteracts network hypersynchrony.^6^ However until recently, this cell-type specific effect of TAU ablation hadn’t been demonstrated in vivo. Here, Shao and colleagues used an established DS model (Scn1a^RX/+^ which results in Na_V_1.1 haploinsufficiency) combined with an elegant genetic strategy to either selectively ablate tau in excitatory or inhibitory neurons, or to obtain a ubiquitous TAU downregulation using antisense oligonucleotides.^7^

The authors first corroborated that TAU ablation only yields resistance to chemically induced epileptic activity when ablated from excitatory, but not inhibitory neurons. Furthermore, only the targeted TAU ablation on excitatory neurons improved survival, reduced network hyperexcitability, and ameliorated some social and behavioral abnormalities in a DS mouse model, in line with previous reports.^5^ Next, the authors examined the PI3K-AKT-mTOR pathway, an overactivated pathway in epilepsy syndromes and an attractive therapeutic target. In contrast to the previous authors’ genetic ablation studies,^4^ restricted TAU ablation in excitatory neurons was only able to restore the increased relative pAKT/total AKT ratio in DS mice but was not sufficient in restoring pS6/totalS6 ratio.^7^

In order to test a more readily translatable TAU reduction strategy for DS, Shao and colleagues targeted the *Mapt* transcript, coding for TAU protein, with mRNA-targeting antisense oligonucleotides (TAU-ASOs) to stably reduce mRNA and TAU protein expression. In line with their cell-specific ablation strategy, TAU-ASOs were able to improve survival, reduce network hyperexcitability, and ameliorate social and behavioral abnormalities exhibited in the DS model, presumably due to a pronounced reduction of TAU across various brain regions involved in seizure onset. This observation reinforces the idea that endogenous tau is an excitability modifier, or possibly an enabler of hyperexcitability-promoting mechanisms.^4,6,8^ In contrast to cell-specific TAU ablation, the use of a ubiquitous TAU-ASO also restored pS6/totalS6 ratio to wild-type levels, suggesting the former strategy may not target all the relevant cell populations (i.e., glial cells) to restore PI3K-AKT-mTOR hyperactivation or that both excitatory and inhibitory neurons need to be targeted for a full rescue.^7^

This is the first study to utilize a cell-specific ablation strategy for TAU *in-vivo*, and interestingly the first TAU-ASO strategy to treat a genetic model of epilepsy with a completely unrelated etiology (Nav1.1 haploinsufficiency affecting inhibitory neurons). Considering that epilepsies and tauopathies are emerging as two closely related pathologies, Shao and colleagues support a growing dogma that endogenous TAU may be modulated to decrease hyperexcitability in pathologies without a direct TAU implication.^2-6^ In this case, TAU reduction decreases the excitability of excitatory neurons without any direct effect on the Nav1.1 loss affecting inhibitory neurons. This has importance for other diseases where early hyperexcitability may be driving pathology, including other genetic and acquired epilepsy syndromes, early phases of Alzheimer’s Disease,^9^ and Autism Spectrum Disorder.^4^

Although this study exemplifies a novel excitability-modifying strategy, complete phenotypic recovery was not achieved. This may be attributed to the delivery of TAU-ASOs starting at postnatal day 10, where cortical circuits may already be undergoing maladaptive remodeling.^7^ Importantly, disease phenotype reversibility is achievable after symptom onset through restoration of Nav1.1 function^10^ and may represent a more translatable treatment considering DS patients don’t exhibit any major symptoms before a first febrile seizure. In this study, a full rescue of the epileptic and cognitive phenotypes has been achieved with Nav1.1 restoration at P30, and the suppression of seizures have been even observed when Nav1.1 was restored at P90.^10^ In addition, although global TAU reduction strategies are generally well tolerated, TAU has multifaceted functions and there are cautions to consider with regards to altering synaptic plasticity mechanisms.^8^ Indeed, the use of viral vector technology offers more selective and regulatable strategies that can deliver TAU-lowering therapeutics to specific maladaptive circuitry and specific neuronal populations, as suggested by the data in excitatory neurons, which may produce fewer off-target effects.

In conclusion, despite the fact that approaches seeking to enhance SCN1A gene expression/restore Nav1.1 function are already in clinical trials,^1^ Shao and colleagues have uniquely demonstrated the therapeutic efficacy of TAU-lowering therapeutics in neurodevelopmental disorders characterized by hyperexcitability. This opens an exciting new avenue of treatment for patients with neurodevelopmental encephalopathies, with the near future possibility of a unique TAU therapy for many different etiologies.
